# Carcinosarcoma of the Bladder: A Case Report and Review of the Literature

**DOI:** 10.1155/2013/716704

**Published:** 2013-07-15

**Authors:** Doğan Atılgan, Yusuf Gençten

**Affiliations:** Department of Urology, Gaziosmanpaşa University School of Medicine, 60100 Tokat, Turkey

## Abstract

Carcinosarcoma of the urinary bladder is a rare neoplasm that is composed of malignant epithelial and mesenchymal components. In these tumors, histogenesis and biological behaviour remain controversial. Approximately 70 cases have been reported in the literature, usually as case reports or a small series. A series of 221 cases using the Surveillance, Epidemiology and End Results (SEER) Program database has been reported recently. Optimal treatment is uncertain. Herein, we report a case of sarcomatoid carcinoma of urinary bladder of a farmer aged 84 years old with a year history of hematuria and dysuria. A transurethral resection of the tumor (TUR-T) revealed a carcinosarcoma. The patient underwent radical cystectomy, and there is no tumor recurrence for 15 months after treatments.

## 1. Introduction

Sarcomatoid carcinoma or carcinosarcoma (CS) is a rare neoplasm of the urinary bladder that is defined by the World Health Organization as a biphasic tumor consisting of malignant epithelial and mesenchymal components [1]. Approximately 70 cases have been reported in the literature mostly as a case report or limited series [2]. The carcinosarcoma of the bladder is more commonly seen in males, and male/female ratio is nearly 2 : 1. The disease appears in the seventh decade of life [3].  The most common symptoms are macroscopic hematuria and dysuria. Generally more than 70% of cases present with advanced stage and have a worse prognosis than conventional urothelial carcinomas [3]. The mesenchymal element of carcinosarcoma lacks epithelial markers [2, 4], and patients with carcinosarcoma present at a more advanced stage and are at greater risk for death compared to patients with high-grade urothelial carcinoma [3]. In cases of bladder carcinosarcoma, evidence supporting a monoclonal origin for the epithelial and mesenchymal components was revealed using loss of heterozygosity studies with microsatellite markers [5, 6], while there exists a hypothesis that multiclonal stem cells of the epithelial and mesenchymal components play a causative role. It is also known that bladder carcinosarcoma develops following cyclophosphamide therapy [5] as well as radiotherapy to the bladder [7]. Here we report a case of bladder carcinosarcoma which was more than 10 cm in diameter.

## 2. Case Presentation

**Figure 1:**
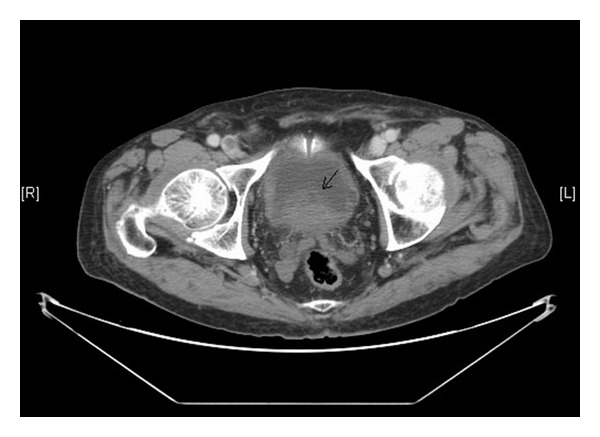
Computed tomography revealing a 5 cm size heterogeneous mass in the right wall and base of the bladder.

**Figure 2:**
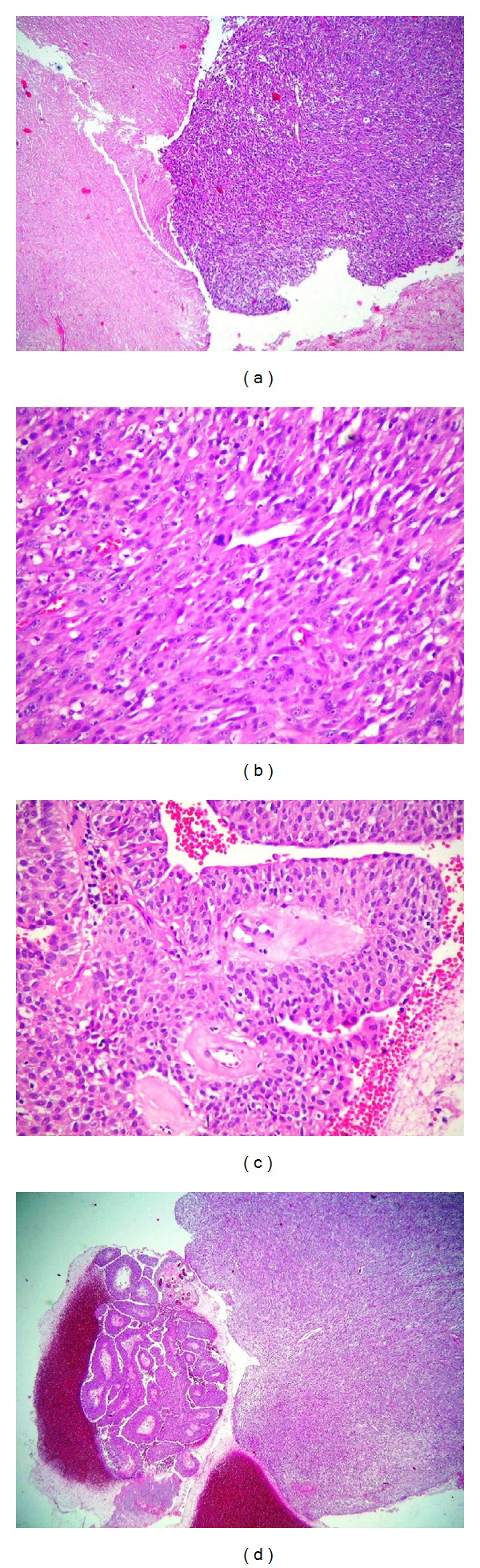
(a) Microscopic overview of the malignant urothelial tumor with epithelial and sarcomatoid components (H-E, ×5). (b) Papillary urothelial carcinoma (H-E, ×30). (c) Sarcomatoid component of the tumor showing pleomorphic, spindle cells. There is atypical mitotic figure in centre of tumor (H-E, ×30). (d) Large size of necrosis in sarcomatoid tumor (H-E, ×10).

A 84-year-old man presented with gross hematuria and dysuria. There were no abnormalities in the physical examination and on biochemical parameters. Pelvic CT revealed a 5 cm heterogeneous mass in the right wall and base of the bladder (Figure 1). Cystoscopy revealed massive smooth polyp with papillary area on the posterior wall of bladder. Therefore, transurethral resection of the bladder was performed. Histology of the tumor was diagnosed as carcinosarcoma composed of biphasic malignant epithelial and mesenchymal elements (Figure 2). Tumor is large and polypoidal with surface necrosis and deep muscle invasion of the bladder wall. With these findings the patient underwent radical cystoprostatectomy and ileal conduit operation. Additionally, pelvic lymph node dissection was performed. Histopathological examination of the specimen revealed that tumor was diagnosed as carcinosarcoma and the tumor had invaded the lamina propria, but it had not invaded the muscle. After an uneventful early postoperative period, the patient, biochemical parameters, and imaging findings were good in the follow-up controls, 15 months postoperatively.

## 3. Discussion

The histological features of CS of the bladder may vary. Macroscopically, these tumors are usually large, polypoid, or nodular. The most reported cases contain high-grade papillary/undifferentiated urothelial carcinoma. The other subtypes with epithelial origins like small-cell carcinoma, squamous carcinoma, and adenocarcinoma have been reported. The most common sarcomatous elements are chondrosarcoma, leiomyosarcoma, and malignant fibrous histiocytoma. In our case the sarcomatous element was a mixture of chondrosarcoma and osteosarcoma [3]. In 1856, Ordonez recorded the first report of a malignant bladder tumour containing elements of cartilage or bone [8].

The etiology of sarcomatoid tumors is unclear, but history of previous radiotherapy or chemotherapy may lead to bladder disorders, and this also causes the formation of sarcomatoid carcinoma. The most typical age at diagnosis is 60 to 80 years, but some studies suggest a range from 41 to 96 years. The majority of patients (89.1%) were white [2].

Kikuchi and colleagues suggested that the frequent location of these tumours in the trigone is evidence of an origin from the Wolffian body [9]. In recent studies, the most common location of carcinosarcomas was the lateral wall of the bladder [2]. In our case, the tumour was located at the trigone of bladder. In most cases, patients present with symptoms similar to urothelial bladder malignancy, such as hematuria. Other common symptoms include dysuria, frequent urination, and obstructive symptoms. Our patient presented with hematuria.

Carinosarcoma of the urinary bladder is very aggressive neoplasms, and there is no standard treatment for this disease. A variety of treatment modalities have been described, but optimal treatment requires rather a multimodality therapy [3]. The only curative management of this kind of neoplasm could be early detection and aggressive surgery [10]. Transurethral resection and partial cystectomy carry the risk of incomplete tumor resection [2, 3]. Radical cystectomy may be an effective treatment option for both superficial and deeply invasive disease [11]. Radical cystectomy with pelvic lymphadenectomy appears to be the main treatment of this disease [2, 3]. Despite this approach, local recurrence and/or metastasis rates were very high after radical surgery [3]. The other preferred modalities of treatment include cystectomy or TURB with or without radiation therapy and chemotherapy [10]. Furthermore, chemotherapy and radiotherapy do not provide apparent survival advantages [10]. Radiotherapy or chemotherapy has yielded conflicting results. Neoadjuvant/adjuvant radiochemotherapy has been used in many cases, and there were complete responses after neoadjuvant treatment [12]. Tazi et al. reported that neoadjuvant radiochemotherapy with radical cystectomy was provided 20 months of recurrence-free survival [13].

CS has a poor prognosis despite of all treatment modalities and median cancer-specific survival approximately for 14 months. The significant prognostic factor was tumor stage for cancer-specific survival in multivariate analyses. Cancer-specific survival was significantly better for those who underwent cystectomy instead of transurethral resection. In a recent large retrospective study which analyzed 221 cases, the overall 5-year cancer-specific survival rate after radical cystectomy was 20.3%. The 1-, 5-, and 10-year survival rates for carcinosarcoma of the urinary bladder were 53.9%, 28.4%, and 25.8%, respectively [2]. In present case, patient has survived without recurrence for 15 months.

## 4. Conclusion

Although CS of the bladder is very rare, aggressive, and lethal tumor, disease-free survival times could be extended with radical combination therapies. Further studies with a number of cases and longer follow-up periods are needed to illuminate clinical significance and prognosis of this disease, as well.
